# Identification of novel therapeutic targets for ovarian hypofunction through hormonal pathway regulation

**DOI:** 10.1186/s12896-026-01110-8

**Published:** 2026-03-07

**Authors:** Yunqi Wang, Lijuan Xiang, Rui Zhao, Xiao-Juan Zhang, Yang Chen

**Affiliations:** 1 Department of Gynecology and Obstetrics, Guangdong Provincial Hospital of Chinese Medicine Hainan Hospital (Hainan Traditional Chinese Medicine Hospital), Heping Road, Meilan District, Haikou, 570100 China; 2 Department of TCM, Hainan Women and Children’s Medical Center, Longkun Road, Qiongshan District, Haikou, 571100 China

**Keywords:** Ovarian hypofunction, Follicle-stimulating hormone receptor, Mendelian randomization, Systems biology, Multi-epitope peptide candidate

## Abstract

Ovarian hypofunction (OH) is a multifactorial endocrine disorder characterized by impaired folliculogenesis, hypoestrogenism, and subfertility, affecting an estimated 10–12% of reproductive-aged women worldwide and up to 1% of women under 40 years of age. Current interventions, including hormone replacement therapy (HRT), gonadotropin stimulation, and assisted reproductive technologies (ART), remain largely symptomatic, carry safety concerns, and fail to restore intrinsic ovarian activity. To address this unmet need, we employed an integrative computational strategy combining systems-level biological insights with immunoinformatics approaches to identify and evaluate a multi-epitope peptide candidate targeting the follicle-stimulating hormone receptor (FSHR). Seven FSHR-derived peptides with high predicted immunogenic potential were identified, validated for non-allergenicity and non-toxicity, and fused with flexible linkers into a 62-amino-acid construct. Physicochemical analysis indicated stability (instability index 38.80), thermostability (aliphatic index 89.68), hydrophilicity (GRAVY −0.365), and favorable solubility (Protein-Sol 0.584 compared with an *E. coli* average of 0.45). Codon optimization improved the codon adaptation index from 0.64 to 0.90 and reduced GC content from 63.98% to 53.23%, suggesting high expression potential in *E. coli*. Molecular docking with TLR2 yielded a favorable docking score (−296), supported by extensive hydrogen bonding and hydrophobic interactions. Long-timescale molecular dynamics simulations (500 ns) supported structural stability, with RMSD plateauing at approximately 0.3 nm, a compact radius of gyration (2.52–2.55 nm), and a persistent hydrogen-bond network (1250–1400 bonds). In silico immune simulations suggested balanced humoral and cellular immune responses, characterized by early IgM production, class switching to IgG, IL-2–associated T-cell expansion, and immune memory formation. Overall, this computational study identifies a structurally stable and immunologically promising peptide candidate for ovarian hypofunction, providing a rational basis for future experimental validation.

## Introduction

Ovarian hypofunction (OH) is a multifactorial endocrine abnormality characterized by reduced ovarian activity and inefficient folliculogenesis, resulting in inadequate production of major reproductive hormones such as follicle-stimulating hormone receptor (FSHR) ligands, estrogen, and progesterone. Such hormonal imbalance impairs the maturation of ovarian follicles and ovulation, ultimately leading to menstrual disturbances, anovulation, or infertility in affected individuals [1]. Patients commonly report oligomenorrhea or amenorrhea, low libido, hot flashes, and mood lability, and in some cases develop premature ovarian insufficiency. Beyond reproductive consequences, ovarian hypofunction is associated with metabolic dysregulation, reduced bone mineral density, and increased cardiovascular risk related to hypoestrogenism [2]. Its molecular etiology is complex and multifactorial, involving genetic susceptibility, autoimmune components, and environmental influences; therefore, despite its high clinical relevance, effective disease-modifying therapies remain limited [3].

Worldwide, ovarian hypofunction and its associated conditions, including diminished ovarian reserve (DOR) and premature ovarian failure (POF), affect a growing proportion of reproductive-aged women. Epidemiological data suggest that approximately 10–12% of women may experience some form of ovarian dysfunction during their lifetime, with premature ovarian insufficiency reported in about 1% of women under 40 years of age and up to 0.1% of those under 30 years [4]. This burden is particularly pronounced among women of advanced maternal age and those exposed to chemotherapeutic agents, environmental toxins, or autoimmune insults. OH not only reduces fecundity but also compromises the success of assisted reproductive technologies (ART) due to poor oocyte quality and diminished ovarian responsiveness to stimulation [5]. The socioeconomic and psychological burden is substantial, as affected women often undergo multiple ART cycles and experience long-term health consequences associated with chronic estrogen deficiency [6].

The follicle-stimulating hormone receptor (FSHR) is a G protein-coupled receptor predominantly expressed on granulosa cells of ovarian follicles and plays a central role in follicular growth, estrogen biosynthesis, and follicle maturation through FSH-mediated signaling pathways. Given its essential role in ovarian physiology and its relevance to ovarian hypofunction, FSHR was selected as the target protein in this study for computational evaluation and peptide-based construct design. This selection was based on its functional importance, target specificity, and suitability for epitope-oriented immunoinformatics analysis [7]. Accordingly, we employed a computational immunoinformatics workflow to design and evaluate a multi-epitope construct targeting FSHR, with emphasis on predicted antigenicity, safety, structural stability, and immune response characteristics.

At present, ovarian hypofunction is managed primarily with hormone replacement therapy (HRT) and, in selected cases, gonadotropin stimulation or oocyte cryopreservation. Although HRT can alleviate symptoms of hypoestrogenism, it does not restore intrinsic ovarian function or follicular recruitment and is associated with risks such as thromboembolism and hormone-dependent malignancies [8]. ART strategies relying on gonadotropin stimulation often yield suboptimal outcomes in patients with OH due to ovarian insensitivity. Emerging regenerative approaches, including stem cell therapy and platelet-rich plasma (PRP) infusion, have been explored, but remain costly and lack robust clinical evidence supporting long-term efficacy and safety [9]. Collectively, these limitations highlight a critical need for mechanistically informed therapeutic strategies that target the molecular pathways governing folliculogenesis and ovarian endocrine regulation [3].

Advances in computational biology have facilitated the integration of genetic data with systems-level analyses to investigate molecular mechanisms underlying complex disorders, including ovarian hypofunction. Such approaches enable the prioritization of biologically relevant targets and the exploration of regulatory networks involved in ovarian hormone signaling [10]. Following target identification, in silico immunoinformatics, peptide engineering, and molecular modeling approaches can be applied to develop and refine candidate constructs in a cost- and time-efficient manner compared with conventional experimental screening [11]. These computational strategies are particularly valuable for ovarian disorders characterized by molecular heterogeneity, which has historically limited direct experimental validation. Consequently, systematic in silico pipelines offer a rational framework for identifying hormone-regulating proteins and designing peptide-based candidates with minimized off-target effects [12].

In this study, we aimed to design and computationally evaluate a peptide-based candidate targeting the follicle-stimulating hormone receptor (FSHR), a key regulator of folliculogenesis and ovarian endocrine signaling. The objective was to assess the predicted immunogenicity, safety, structural stability, and receptor interaction potential of a multi-epitope construct relevant to ovarian hypofunction. All analyses were conducted exclusively in silico, and the findings are interpreted strictly as predictive computational evidence rather than experimental validation. This work provides a rational framework for future experimental studies to evaluate specificity, safety, and functional endocrine outcomes.

## Methodology

A peptide candidate was developed using a comprehensive **immunoinformatics-based** approach. **To predict peptides, assemble the construct, evaluate physicochemical and structural properties, and assess immune-related features**, a variety of bioinformatics tools and **web-based** servers were employed. **Each step was systematically performed** to evaluate the predicted immunogenic potential, stability, and safety of the proposed construct.

### Retrieval and selection of target proteins

Protein sequences of target proteins were retrieved in FASTA format (https://www.uniprot.org/) of the UniProt database. The UniProt provides an extensive database of protein sequence as well as function data and this marks it as the initial source of peptide candidate design [13].

### Peptide prediction

Potential immunogenic peptides have been retrieved with the help of AMP pred Database (https://aps.unmc.edu/tools) which assesses the probability of a peptide to **elicit an immunological response**. Peptides with **higher prediction scores** were shortlisted for further analysis [14].

### Peptide validation

The set of candidate peptides then were evaluated again through allergenicity AllerTOP v2.0 platform (https://www.ddg-pharmfac.net/AllerTOP/), and toxicity was analyzed via the ToxinPred tool (https://webs.iiitd.edu.in/raghava/toxinpred/) [15].

### Peptide candidate formation

An appropriate peptide linker was incorporated to connect the selected peptides **to enhance flexibility and ensure proper epitope presentation**. Peptides were linked using an SSL linker, and the resulting construct was **subsequently evaluated** for toxicity, **allergenicity**, and antigenicity [16].

### Immunological and biochemical profiling of peptide candidate

To evaluate the construct’s potential to induce an immune response, antigenicity was assessed using the VaxiJen server. AllerTOP v2.0, which employs auto cross-covariance analysis to classify proteins as allergenic or non-allergenic, was used to predict allergenicity. ToxinPred was utilized to assess toxicity and ensure that the construct did not contain harmful motifs. Protein solubility was predicted using Protein-Sol (https://protein-sol.manchester.ac.uk/), which estimates solubility based on amino acid composition to assess heterologous expression feasibility [17].

### Physicochemical properties

The physicochemical characteristics of the Peptide Candidate that was built were calculated using the ProtParam program (https://web.expasy.org/protparam/). Parameters like molecular weight, theoretical isoelectric point (pI), instability index, aliphatic index, and extinction coefficient were used to estimate protein stability, solubility, and suitability for recombinant expression and purification [18].

### Secondary structure prediction

The PSIPRED service (http://bioinf.cs.ucl.ac.uk/psipred/) was used to estimate the Peptide Candidate secondary structure. This tool uses position specific scoring matrices derived from sequence alignments to separate residues into random coils, beta strands, and alpha helices. Analysis of secondary structure provides insights into protein folding, stability, and potential immunogenicity, as reflected by the generated structural profile [19].

### 3D structure prediction

The tertiary (3D) structure of the Peptide Candidate was modeled using AlphaFold 3 (https://alphafoldserver.com/). This deep-learning-based approach predicts protein structures based on inter-residue distances and folding patterns, generating a three-dimensional model that served as a structural framework for downstream analyses, including molecular docking, stability evaluation, and epitope accessibility [20]. The three-dimensional structure of FSHR used in this study was retrieved from the AlphaFold Protein Structure Database as a predicted model rather than an experimentally resolved structure. This AlphaFold-derived model was used as a computational template to support downstream in silico analyses, including structural visualization, epitope localization, and receptor–construct interaction assessment. The use of predicted structures was considered appropriate for hypothesis-generating analyses, while acknowledging that experimentally determined structures may provide higher confidence for translational applications.

### Structural validation

The modeled Peptide Candidate was structurally validated using PROCHECK and the SAVES server v6.1 (https://saves.mbi.ucla.edu/). Ramachandran plots were generated to evaluate backbone stereochemical quality, and ERRAT scores were calculated to assess overall model reliability and identify potential structural inconsistencies. These analyses confirmed the suitability of the predicted structure for subsequent docking and molecular dynamics simulations [21].

### Molecular docking

HDock was used to molecularly dock the Peptide Candidate with pertinent immunological receptors (https://hdock.phys.hust.edu.cn/). The generated docking conformations were evaluated based on docking scores, cluster ranking, and the number and type of hydrogen bonds formed. Docking results were interpreted as relative interaction scores rather than experimental binding affinities, providing insight into potential receptor engagement [22].

### Interaction analysis

LigPlot+ was used to analyze interactions between the docked Peptide Candidate –receptor complexes (https://www.ebi.ac.uk/thornton-srv/software/LigPlus/). This program generated complex two dimensional schematics that clearly depicted the chemical contacts such as hydrogen bond networks, hydrophobic interactions, and significant interacting residues necessary for immune activation and receptor recognition [23].

### Molecular dynamics (MD) simulations

Molecular dynamics (MD) simulation (500 ns) was carried out for the docked peptide–receptor complex by GROMACS 2023 with the CHARMM36m force field. Neutralization of the complex with counter ions and minimization were performed, followed by solvation in a TIP3P water box. NVT and NPT equilibrations were performed to reach a stable temperature (300 K) and pressure (1 bar), then the system was subjected to a 500ns long production simulation using 2 fs time step. Long range electrostatics was calculated with Particle Mesh Ewald and bond constraints were imposed by LINCS. The stability and binding dynamics of the complexes were then further analyzed in post simulation by root mean square deviation (RMSD), root mean square fluctuation (RMSF), radius of gyration (Rg), hydrogen Bonding, principal component analysis (PCA) along with dynamic cross correlation matrix (DCCM) [24].

### Codon optimization and in-silico cloning

EMBOSS Backtranseq was used to reverse transcribe the Peptide Candidate amino acid sequence into a nucleotide sequence. The online Codon Optimization tool (https://www.novoprolabs.com/tools/codon-optimization) was used to optimize codon use for effective expression in Escherichia coli, guaranteeing excellent translational efficiency and stability. In order to facilitate subsequent cloning, expression, and purification investigations, the optimized gene sequence was then inserted into the pET28a(+) expression vector using SnapGene software [25].

### Immune simulation

The C-ImmSim server was used to model the immunological response that the peptide construct induced (https://kraken.iac.rm.cnr.it/C-IMMSIM/index.php). The simulation modeled B-cell activation, CD4+ and CD8+ T-cell responses, immunoglobulin production, and cytokine secretion, including IL-2 and IFN-γ. The establishment of immunological memory was also assessed. Comparative simulations with and without adjuvant were performed to evaluate the potential impact on immune activation and response durability [26].

## Results

### Retrieval of target proteins

**Fig. 1:**
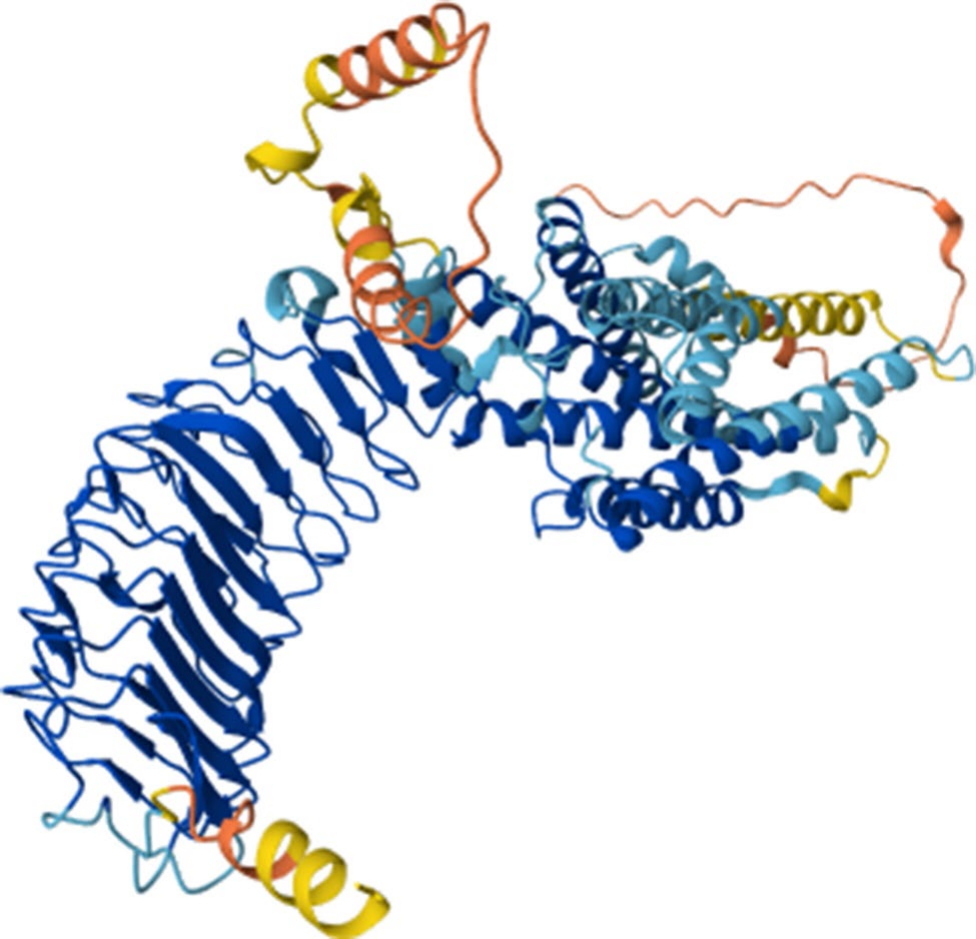
Predicted three-dimensional structure of the follicle-stimulating hormone receptor (FSHR) from *Homo sapiens* (UniProt ID: P23945; Gene: FSHR)

Follicle-stimulating hormone receptor (FSHR) from *Homo sapiens* was identified for analysis (UniProt ID: P23945; Gene: FSHR; Taxonomy ID: 9606). The UniProt record confirms experimental validation at the protein level (PE = 1) and correspondence to sequence version 4 (SV = 4). Fig. 1 shows the predicted three-dimensional structure of FSHR, which serves as a structural foundation for subsequent functional analyses and computational modeling.

### Peptide prediction

Potential immunogenic peptides derived from the target protein were predicted using the CellPPD server, which identifies sequences with a high likelihood of inducing immune responses. Seven peptides were shortlisted based on their prediction scores, and the results are summarized in Table 1.

**Table 1:** Predicted peptides from the target protein

Peptide ID	Peptide Sequence	Prediction Score	Prediction
>1270	TYPSHCCAFA	0.62	AntiCP
>1208	ELPNDVFHGA	0.61	AntiCP
>1272	PSHCCAFANW	0.62	AntiCP
>1280	NWRRQISELH	0.66	AntiCP
>1251	NLKKLPTLEK	0.54	AntiCP

### Peptide candidate formation

**Fig. 2:**
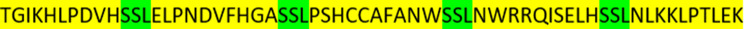
Green indicates linkers, while yellow indicates the selected peptide sequences

A multifunctional construct containing five predicted peptides was successfully assembled to achieve optimal flexibility and structural stability using SSL linkers, as shown in Fig. 2. The construct was predicted to be non-allergenic and non-toxic. The antigenicity score of the proposed peptide candidate was 0.7143, indicating a high predicted antigenic potential.

### Immunological and biochemical profiling of peptide construct

**Fig. 3:**
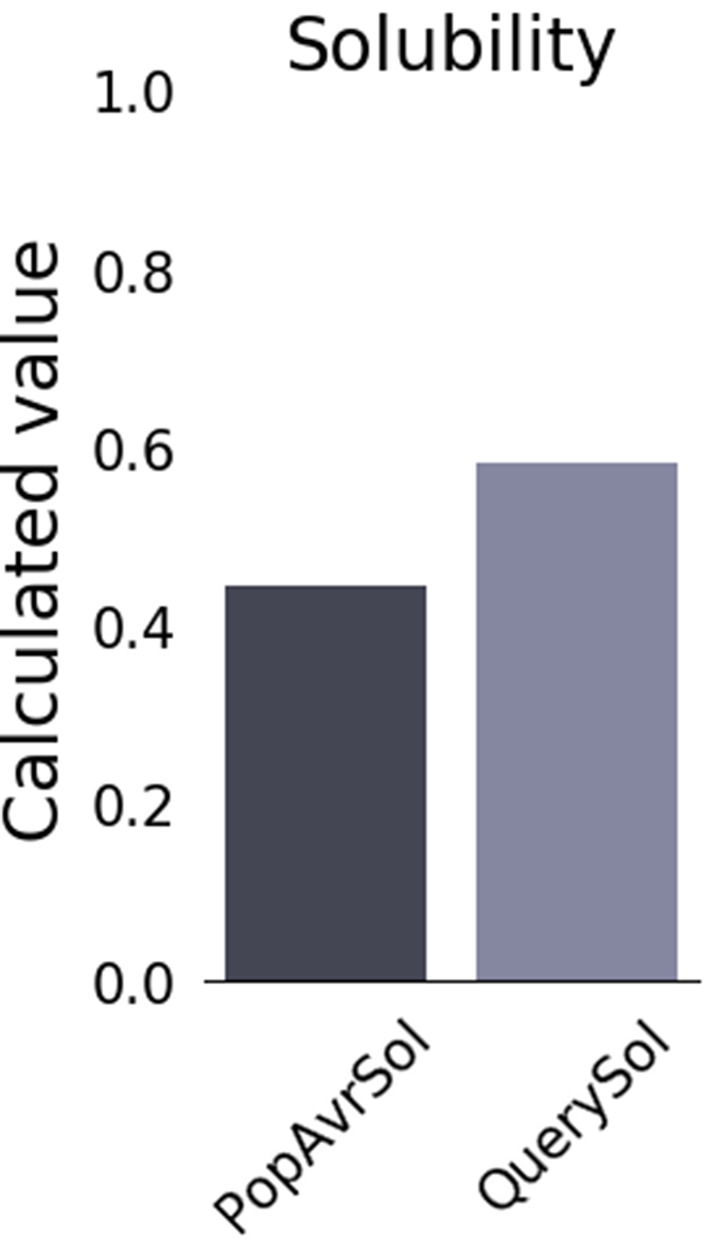
Predicted solubility of the peptide construct (0.584) compared with *E. coli* average solubility (0.45), indicating favorable solubility for expression

Predictions of allergenicity and toxicity supported the favorable safety profile of the final multi-epitope peptide construct by confirming that it is non-allergenic and devoid of toxic motifs. VaxiJen antigenicity screening indicated a high immunogenic potential. Protein-Sol analysis predicted a scaled solubility score of 0.584, suggesting moderate solubility under physiological conditions (Fig. 3). Additionally, the construct was classified as a basic protein, and its suitability for heterologous expression in *Escherichia coli* was supported by a theoretical isoelectric point (pI) of 9.89.

### Physicochemical properties

The designed peptide construct consisted of 62 amino acids with a molecular weight of 6948.90 Da and a theoretical isoelectric point (pI) of 7.84, suggesting a near-neutral to slightly basic character. The sequence contained five negatively charged residues (Asp + Glu) and six positively charged residues (Arg + Lys), indicating a balanced charge distribution. The atomic composition was determined as C₃₀₉H₄₈₃N₈₉O₉₀S₂, comprising a total of 973 atoms. Extinction coefficient analysis at 280 nm predicted values of 11,125 M^− 1^ cm^− 1^ (absorbance 1.601 at 0.1% concentration) under oxidizing conditions and 11,000 M^− 1^ cm^− 1^ (absorbance 1.583) under reducing conditions, indicating moderate absorbance characteristics. The predicted half-life was 7.2 h in mammalian reticulocytes (in vitro), >20 h in yeast (in vivo), and >10 h in *E. coli* (in vivo), suggesting favorable stability across multiple expression systems. The instability index (38.80) classified the construct as stable, while the aliphatic index (89.68) indicated good thermostability. The GRAVY score (−0.365) reflected an overall hydrophilic nature, supporting solubility under physiological conditions.

### Secondary structure prediction

**Fig. 4:**
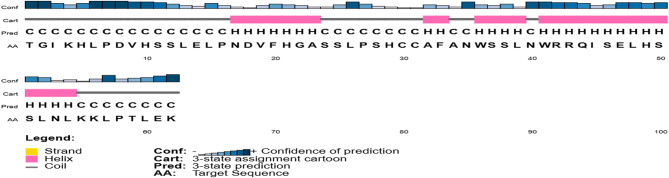
Secondary structure prediction of the target protein generated using PSIPRED. The diagram illustrates predicted α-helices, β-strands, and coil regions along the amino acid sequence, providing insights into the structural organization and potential functional domains

Secondary structure analysis of the designed peptide construct revealed a well-organized distribution of α-helices, β-strands, and random coils. Helical regions (pink) were predominantly located at the N-terminus and core regions, while β-strands (yellow) were interspersed between helices, contributing to overall structural stability. Random coil regions (gray) provided flexibility and facilitated epitope exposure. Several residues were predicted to be extracellular (orange), indicating surface accessibility for immune recognition, and no transmembrane helices were identified, supporting suitability for recombinant production. High confidence scores associated with these predictions suggest reliable secondary structural modeling (Fig. 4).

### 3D Structure Prediction

**Fig. 5:**
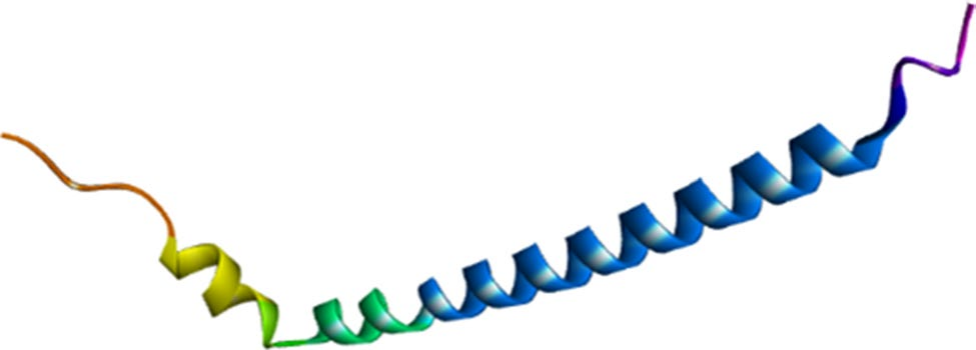
Predicted 3D structure of the designed multi-epitope peptide construct

The three-dimensional structure of the peptide construct was modeled using AlphaFold 3, revealing a compact and well-ordered conformation. The predicted structure contained distinct secondary structural elements, including α-helices and β-strands, with surface-exposed regions corresponding to selected epitopes. This configuration supports epitope accessibility and provides a structural basis for downstream docking and interaction analyses (Fig. 5).

### Structural validation

**Fig. 6:**
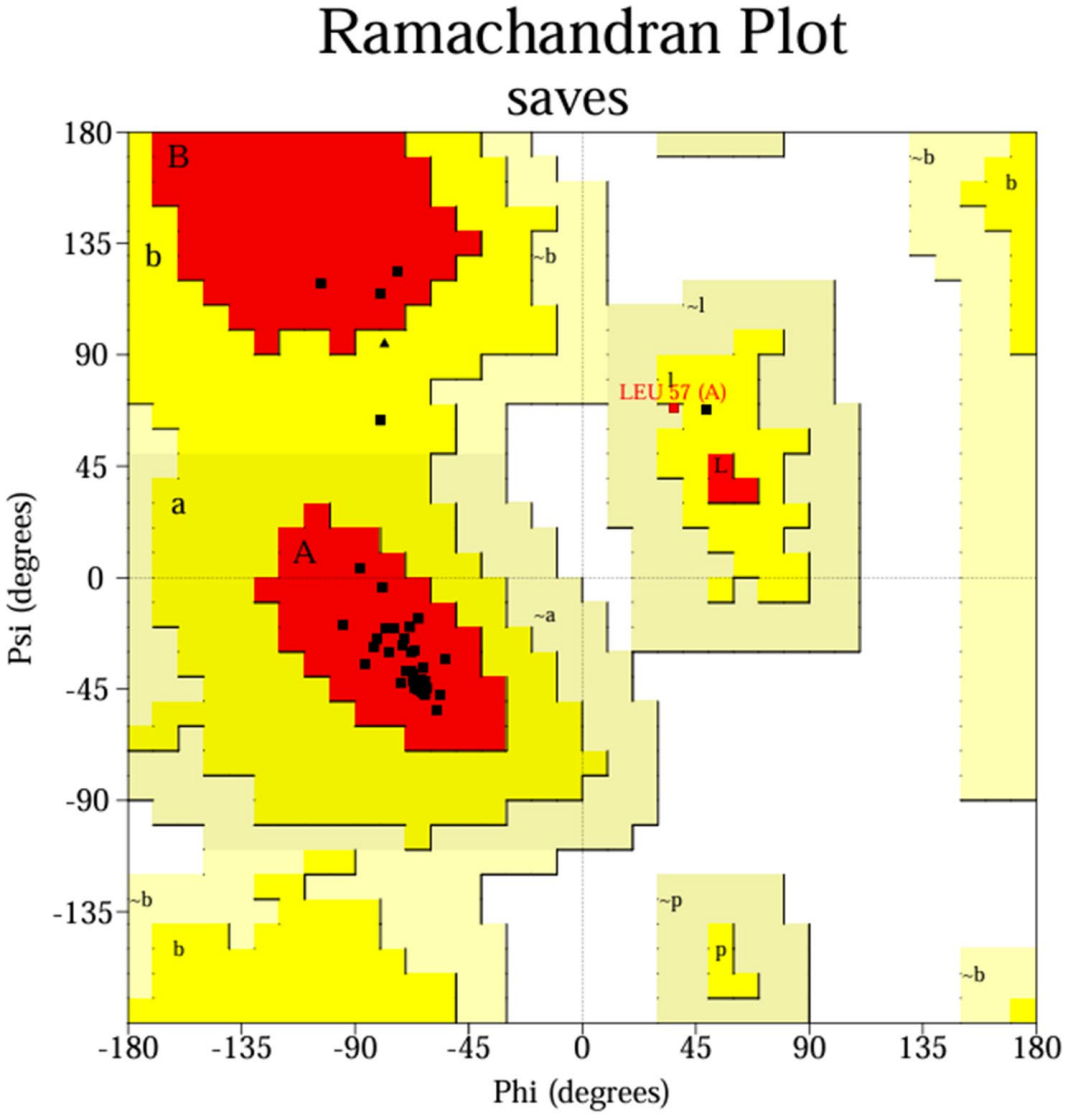
Ramachandran plot analysis of the modeled peptide construct

Structural validation using Ramachandran plot and ERRAT analysis demonstrated high stereochemical quality. Of the 62 residues, 96.3% were located in the most favored regions, with the remaining residues distributed within additionally or generously allowed regions. No residues were present in disallowed regions, confirming acceptable backbone geometry. These results support the reliability of the model for molecular docking and molecular dynamics simulations (Fig. 6).

### Docking and interaction analysis

**Fig. 7:**
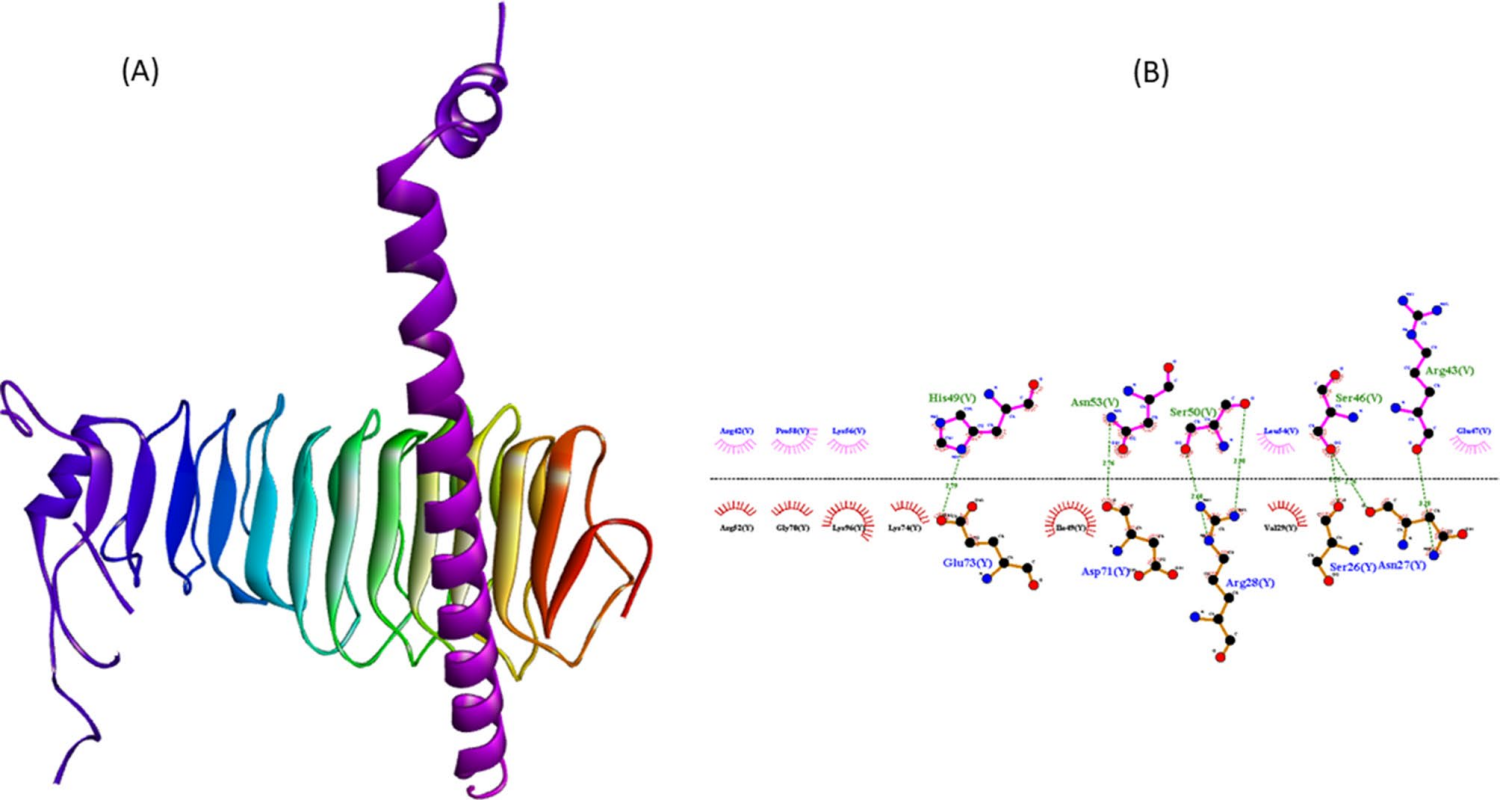
(**A**) three-dimensional representation of the protein–ligand docking complex showing the ligand bound within the active site of the receptor. (**B**) two-dimensional interaction analysis (LigPlot+) illustrating the hydrogen bonds and hydrophobic contacts between the ligand and key receptor residues

Docking of the peptide construct with the TLR2 receptor (PDB ID: 4A92) was performed using the HDOCK server, which reports relative docking scores rather than experimental binding affinities. The top-ranked complex achieved a docking score of −296, suggesting a favorable predicted interaction within the constraints of docking-based scoring. LigPlot+ analysis (Fig. 7) identified key hydrogen bonds and hydrophobic contacts that collectively support interface stabilization and potential innate immune receptor engagement.

### Molecular dynamic (MD) simulations

**Fig. 8:**
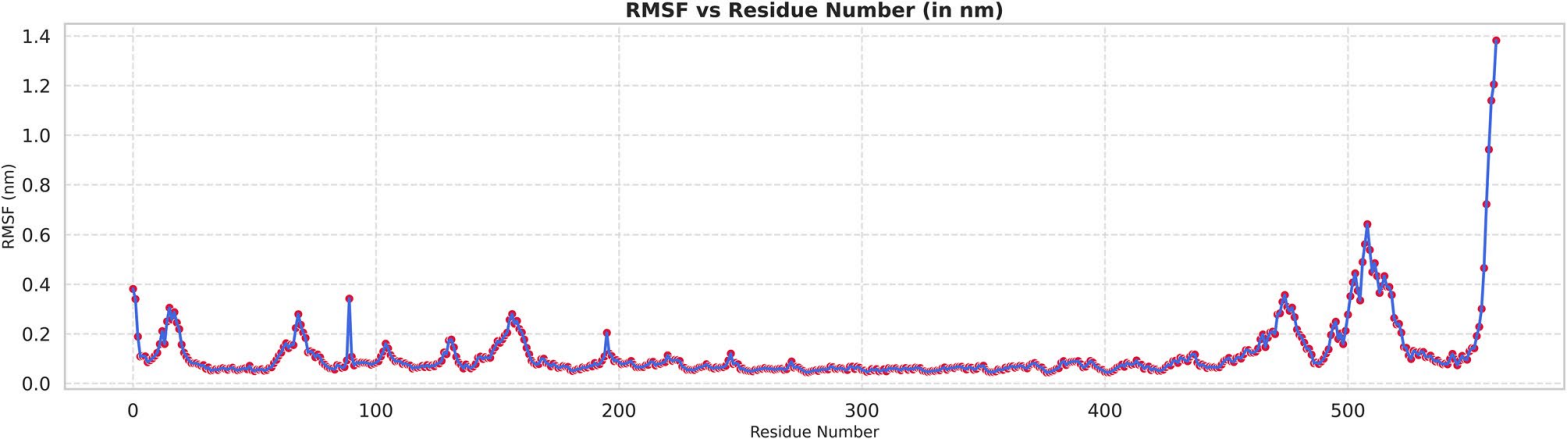
Root mean square fluctuation (RMSF)

The RMSF profile of the peptide-receptor complex in Fig. 8 provides residue-wise information on local flexibility during the 500 ns simulation. The majority of residues within the complex were highly restrained, with fluctuations < 0.3 nm, reflecting a rigid core structure, overall system stability, and inherent flexibility. A few small peaks were occasionally observed in the N-terminal regions and specific loop segments, indicating local flexibility, which is commonly associated with solvent-exposed or disordered regions. The largest increase in RMSF occurred at the C-terminal tail of the peptide chain (>1.2 nm), which is expected, as terminal regions are less structurally constrained and typically more mobile. The interacting interface residues of the peptide candidate and receptor exhibited RMSF values below 0.3–0.35 nm, suggesting that peptide binding resulted in a stable interaction surface without inducing substantial conformational disruption. The low RMSF values observed across the binding interface further indicate that the docked orientation is dynamically compatible, and that the in silico docking did not destabilize the core fold configuration of the receptor.

**Fig. 9:**
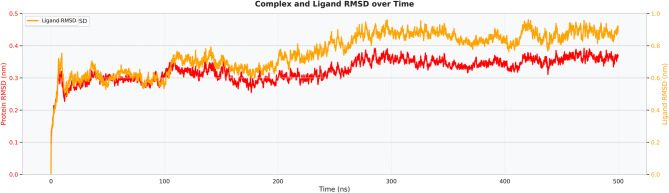
RMSD of the whole complex

The complex RMSD (red curve) as a function of time shown in Fig. 9 reveals a rapid equilibration phase during the first 20–30 ns, followed by stabilization after reaching approximately 0.3 nm. The RMSD exhibited minor oscillations after ~50 ns within the range of 0.28–0.35 nm, indicating that the peptide–receptor complex retained its structural integrity throughout the 500 ns simulation. The absence of any progressive increase or large RMSD spikes beyond 0.35 nm indicates that no significant unfolding or dissociation events occurred. This plateaued RMSD profile supports the conclusion that the docked binding pose was maintained under simulated physiological conditions, reinforcing confidence in the predicted interaction interface.

**Fig. 10:**
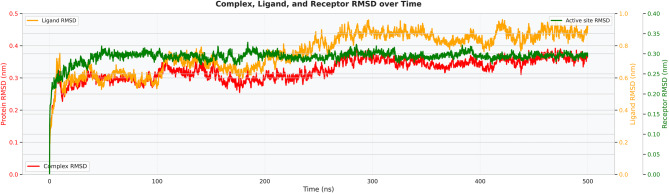
Complex-ligand-receptor combined RMSD

To show the relative stability of each system, a combined RMSD plot was created as shown in Fig. 10 (complex in red, ligand/peptide in orange, and receptor/active site in green). The complex RMSD remained stable at ~0.3 nm, consistent with previous observations, while the peptide RMSD showed slightly higher deviations (~0.4–0.45 nm) yet remained restrained, suggesting moderate flexibility without detachment. The RMSD of the receptor backbone (green) stabilized at 0.28–0.32 nm after equilibration, indicating that the global fold of the receptor did not change substantially upon peptide binding. Most notably, the active-site RMSD closely followed the overall receptor RMSD, with no distortion of the binding pocket observed even during extended simulation times. The concerted behavior of all three RMSD traces indicates a well-anchored complex with localized peptide perturbations but no receptor destabilization.

**Fig. 11:**
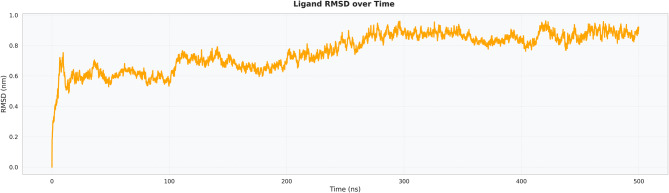
Ligand-Only RMSD

The RMSD between the ligand/peptide and the isolated peptide was higher during the early stages of the simulation (~0.6–0.8 nm) and subsequently stabilized at approximately 0.7–0.8 nm during mid-simulation with minor fluctuations. This greater flexibility relative to the receptor is expected, as the peptide construct includes flexible loops and linker regions (G4S/EAAAK segments). Despite this inherent mobility, the RMSD trace in Fig. 11 reached a stable plateau, indicating that the peptide candidate remained bound to the receptor during conformational breathing rather than adopting a detached state. This adaptability may facilitate epitope exposure and immune recognition while maintaining receptor engagement.

**Fig. 12:**
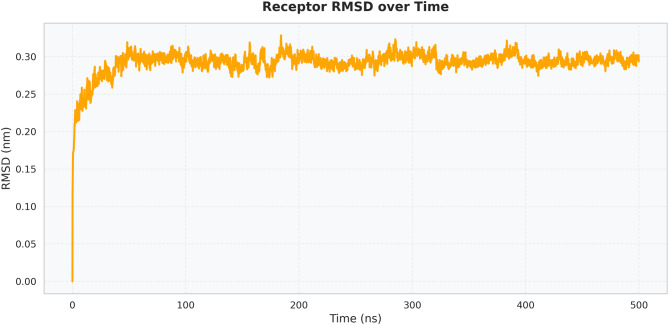
Receptor-only RMSD

The receptor alone presented a highly stable RMSD profile in Fig. 12, reaching ~0.3 nm within the first 20 ns and remaining within a narrow range (0.28–0.32 nm) for more than 60% of the simulation trajectory. This suggests that peptide binding did not induce appreciable unfolding or significant backbone rearrangements in the receptor. The stable RMSD behavior supports compatibility between the designed peptide construct and the immune receptor, in which the complex remains intact without receptor destabilization.

**Fig. 13:**
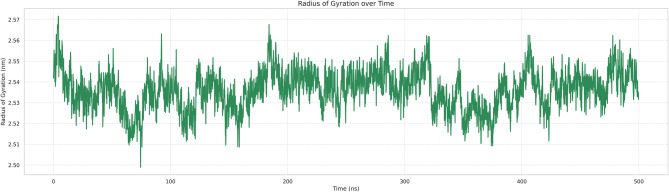
Radius of gyration (Rg)

The moderate Rg profile change shown in Fig. 13 fluctuated slightly around 2.52–2.55 nm during the simulation, implying that a compact and folded conformation of the peptide receptor complex was preserved. Following an initial relaxation phase, during which the Rg slightly decreased over the first ~50 ns, the entire system reached structural equilibrium with minimal evidence of expansion or decompaction. This stable Rg profile further corroborates the RMSD results, suggesting maintenance of a globular structure during long-timescale dynamics.

**Fig. 14:**
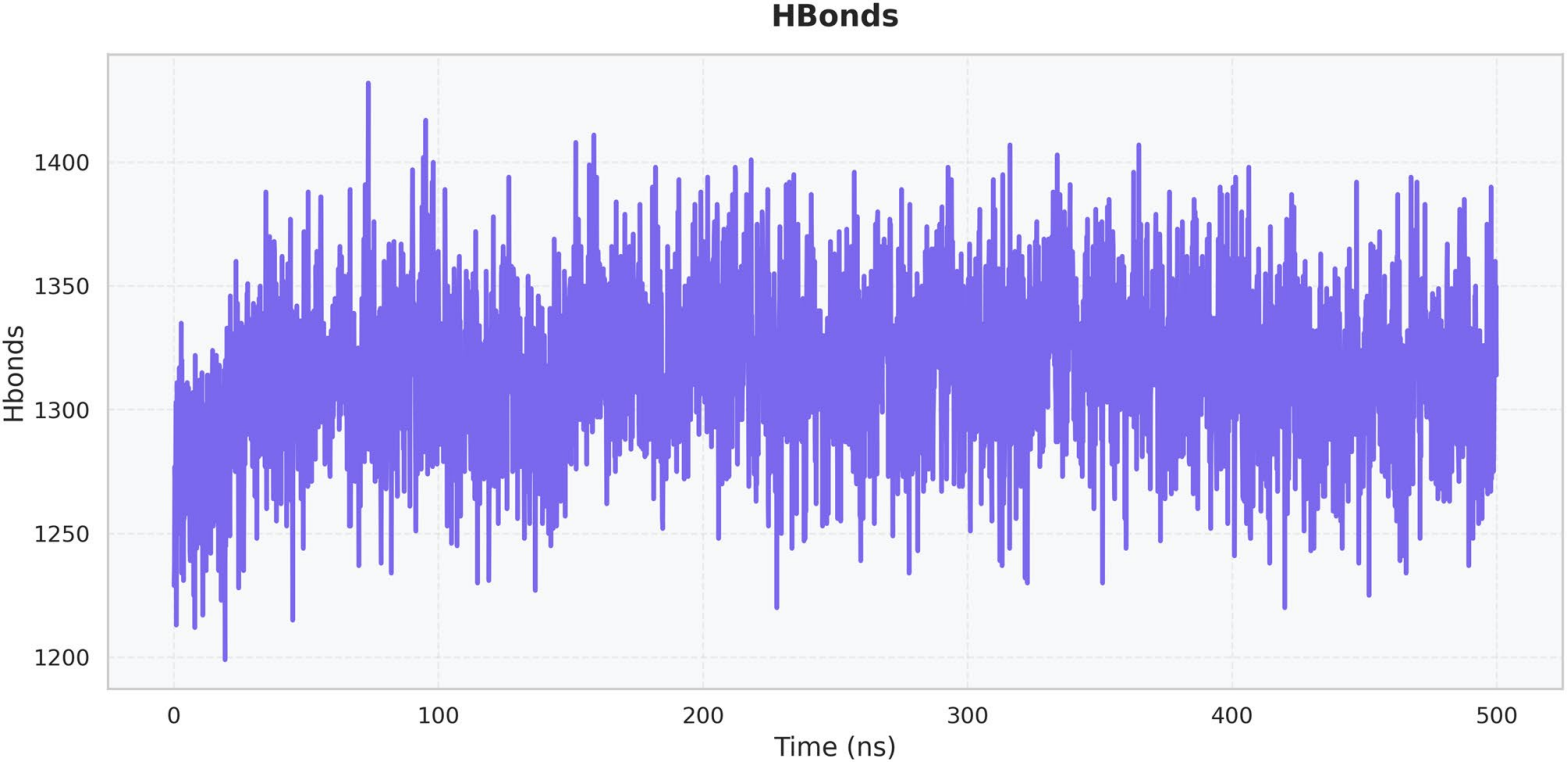
Hydrogen bond analysis

The hydrogen bond plot in Fig. 14 indicates a dense and persistent network of ~1250–1400 hydrogen bonds that survived the 500 ns trajectory. The consistently high number of hydrogen bonds reflects strong intra- and intermolecular stabilization, while regular fluctuations within this range indicate dynamic yet stable bonding interactions. The lack of sharp decay argues against major disruption events. The observed stability and low RMSD values can therefore be attributed, in part, to the maintenance of a robust hydrogen-bonding network at the peptide–receptor interface.

**Fig. 15:**
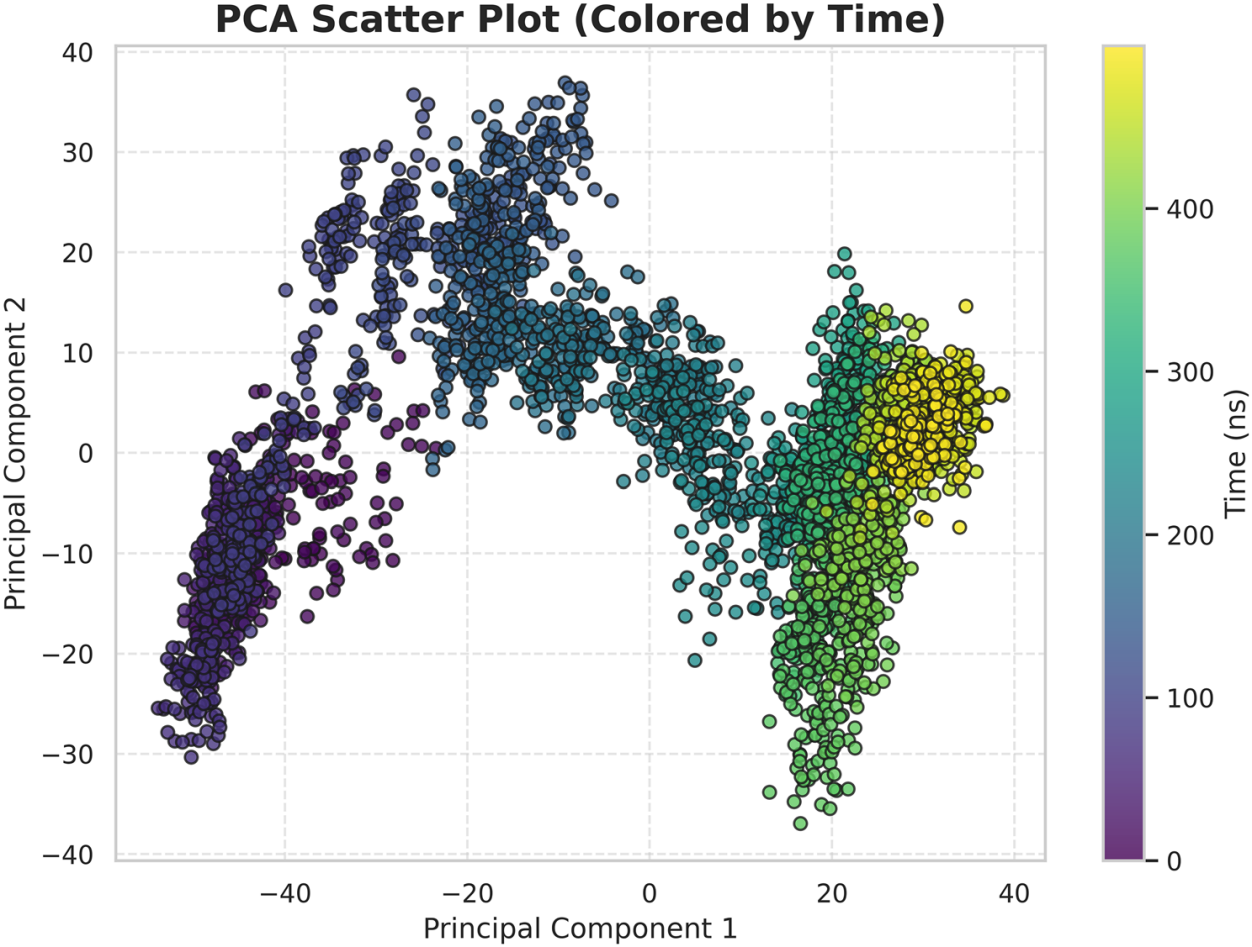
Principal component analysis (PCA)

A PCA scatter plot with simulation time represented as a color scale illustrates the dominant collective motions explored by the complex (Fig. 15). Early simulation frames were more dispersed and subsequently converged into a more confined region around ~150–200 ns, indicating that the system initially sampled a broader conformational space before reaching a stable equilibrium basin. This behavior suggests initial adaptive rearrangements followed by minimal structural variation during the remainder of the simulation and is characteristic of an equilibrated and energetically favorable complex.

**Fig. 16:**
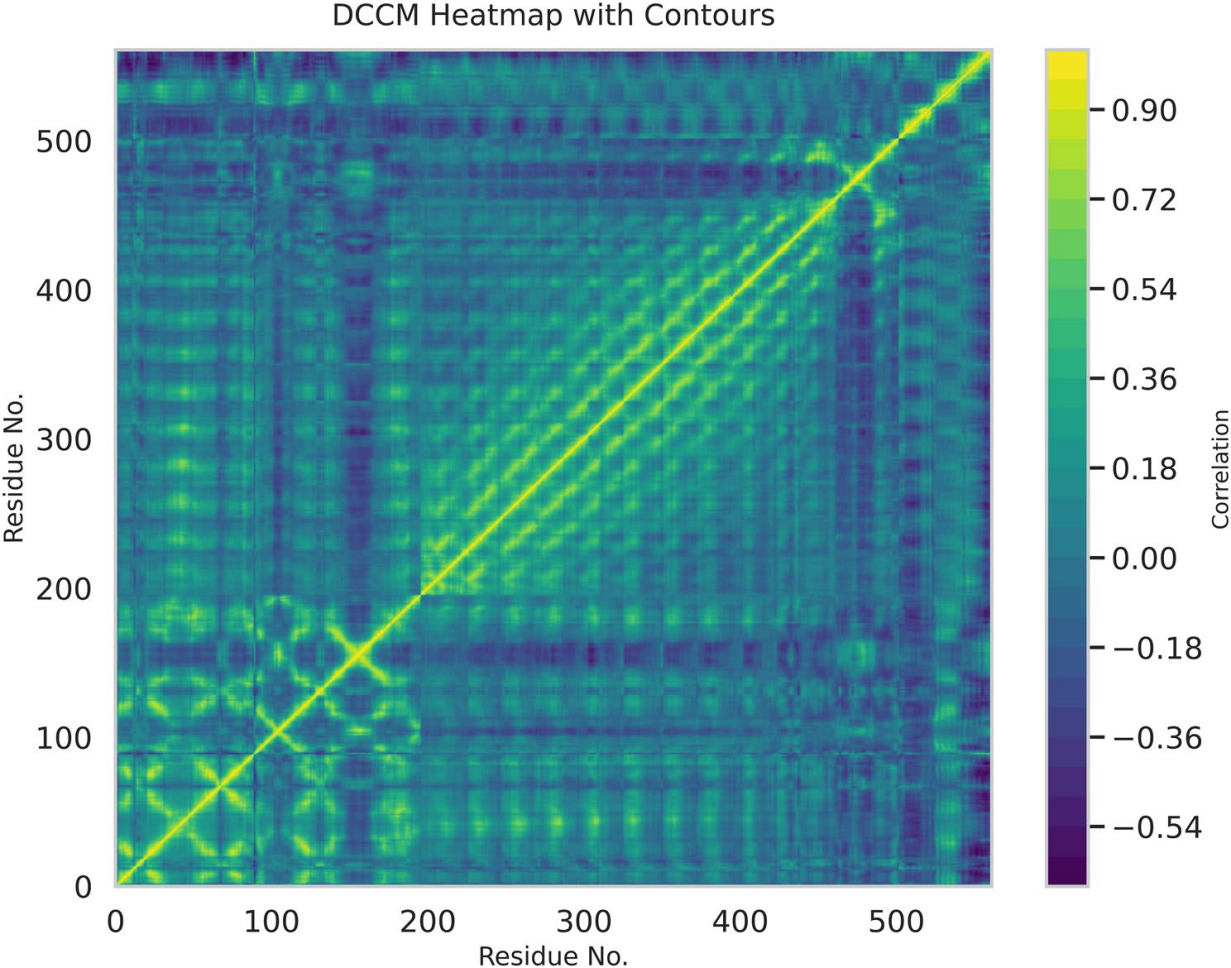
Dynamic cross-correlation Map (DCCM)

A DCCM heatmap in Fig. 16 reveals residue-wise correlated motions within the complex. Strong positive correlations were observed within individual domains of the receptor and peptide core, indicating coordinated movements that support structural stability. Moderate anti-correlated motions were primarily localized between flexible peptide loops and distal receptor regions, reflecting adaptive conformational changes rather than destabilizing effects. The presence of a continuous diagonal line and preserved correlation clusters indicates that the global topology of the complex remained intact, with no large-scale domain separation or disassembly.

### Codon optimization and in-silico cloning

**Fig. 17:**
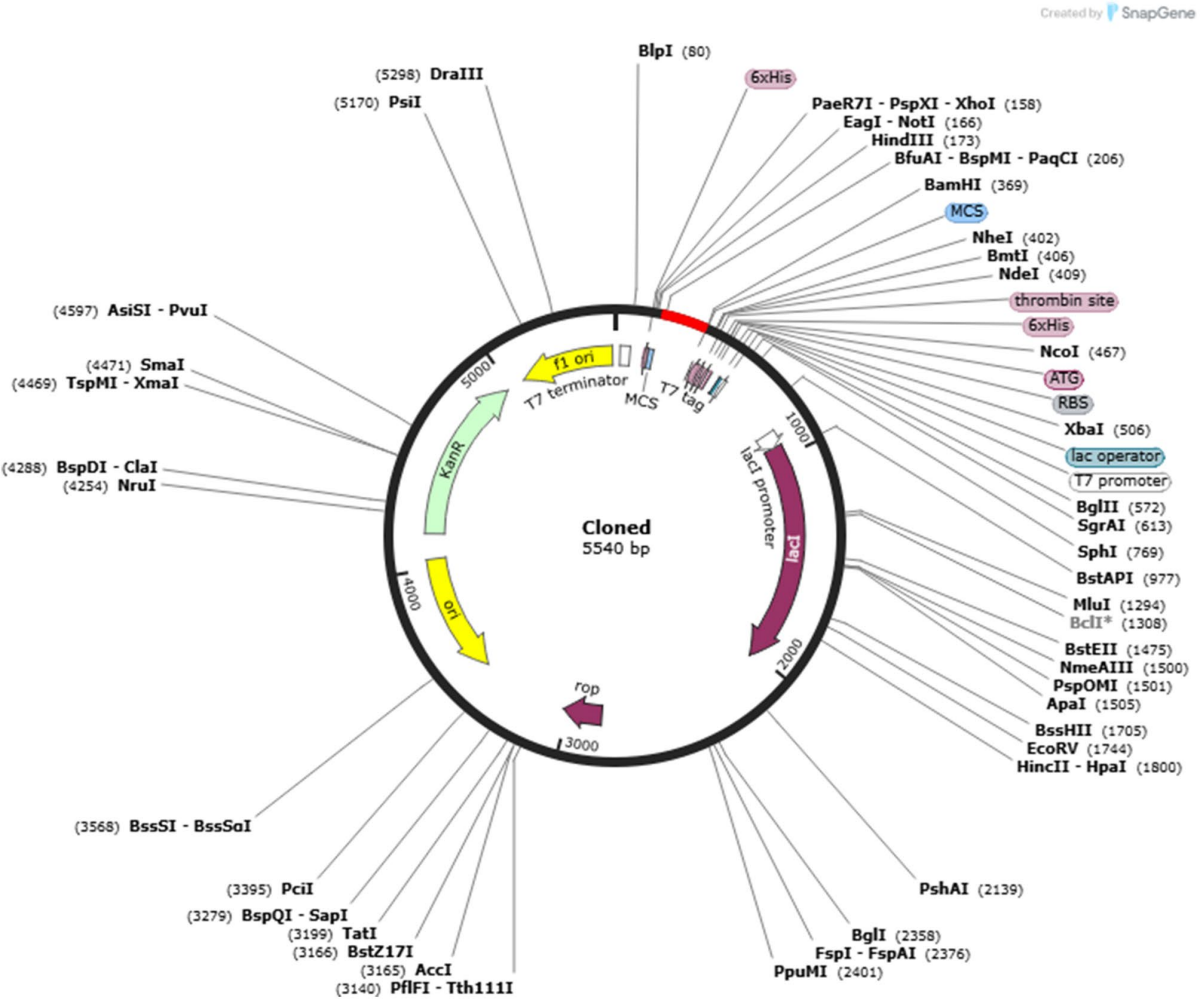
In-silico cloning of the optimized peptide gene into the *E. coli* expression vector pEt28a (+)

Codon optimization improved the Codon Adaptation Index from 0.64 to 0.90, reflecting enhanced compatibility with *E. coli* codon usage. GC content was reduced from 63.98% to 53.23%, minimizing transcriptional inefficiency and secondary structure formation. The optimized gene was inserted into the pET28a (+) expression vector, confirming readiness for experimental expression (Fig. 17).

### Immune simulation

**Fig. 18:**
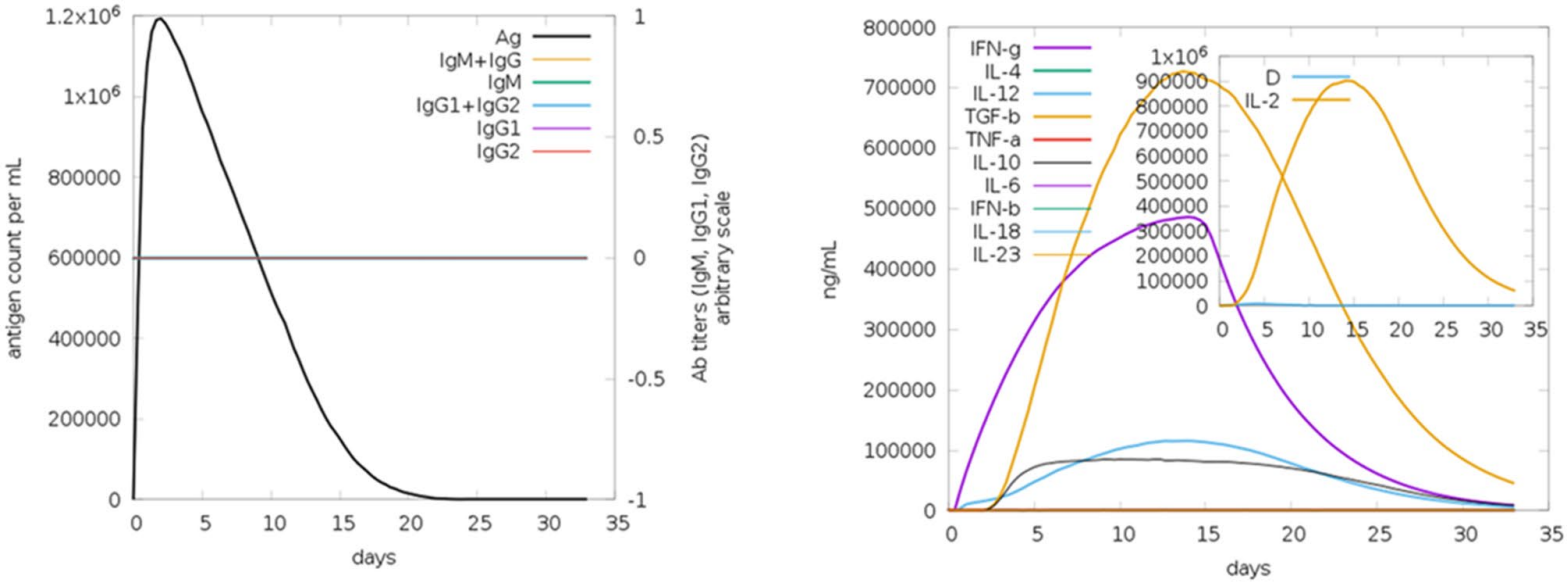
Humoral immune response and cytokine profiling; depicts viral particles, immunoglobulins, and immune complex formation, while cytokine and interleukin concentrations, with IL-2 and danger signals highlighted in the inset

The humoral and cytokine profiles observed for the peptide construct provide further support for its predicted immunogenic potential (Fig. 18). Following an initial immune response characterized by elevated IgM levels, a class switch to IgG isotypes was observed, indicating affinity maturation and the development of long-term B-cell responses. Immune complex formation occurred concurrently with effective antigen neutralization, suggesting functional antibody activity. Cytokine profiling revealed a transient but notable increase in interleukin-2 (IL-2), which is associated with T-cell clonal expansion and the activation of pro-inflammatory cytokine cascades that support Th1-biased immunity. Additionally, the danger signal profile indicated activation of innate immune pathways and effective leukocyte recruitment, as illustrated in Fig. 18.

**Fig. 19:**
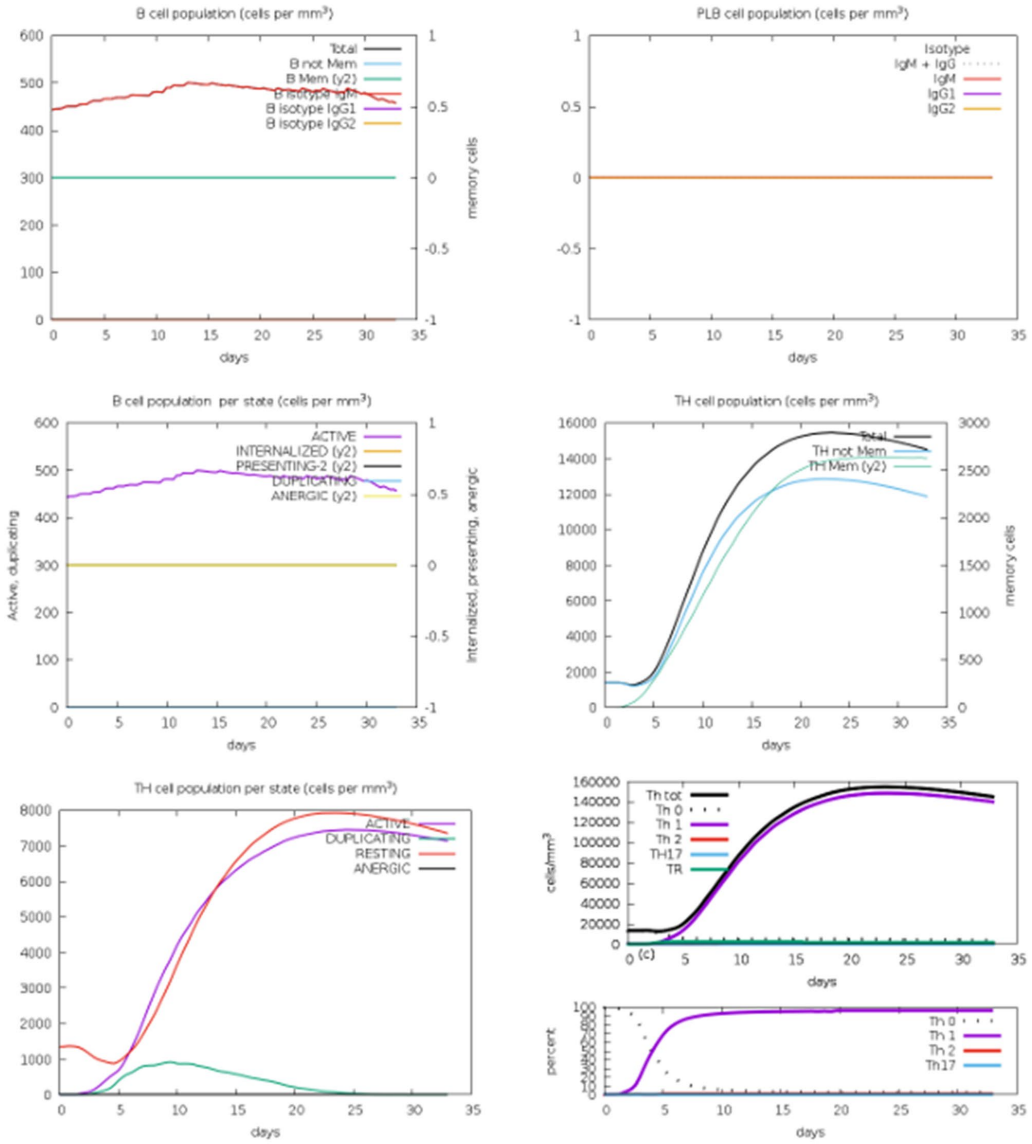
Dynamics of immune cell populations, including activated, internalized, MHC class II–presenting, mitotic, anergic, and resting cells, throughout the simulation

The dynamics of cellular immune responses induced by the peptide construct are presented in Fig. 19. The C-ImmSim simulation demonstrated a significant increase in antigen-presenting cell (APC) populations following antigen exposure. Activated immune cells proliferated, while internalized antigen-bearing cells persisted in processing and presenting antigens via MHC class II molecules. The sustained proportion of mitotically active cells suggested ongoing clonal expansion, whereas the low fraction of anergic and resting cells indicated effective immune activation without tolerance induction. Efficient antigen uptake and presentation to T helper cells, facilitated by the dominance of active and MHC II–expressing populations, supports the initiation of downstream B- and T-cell–mediated immune responses.

**Fig. 20:**
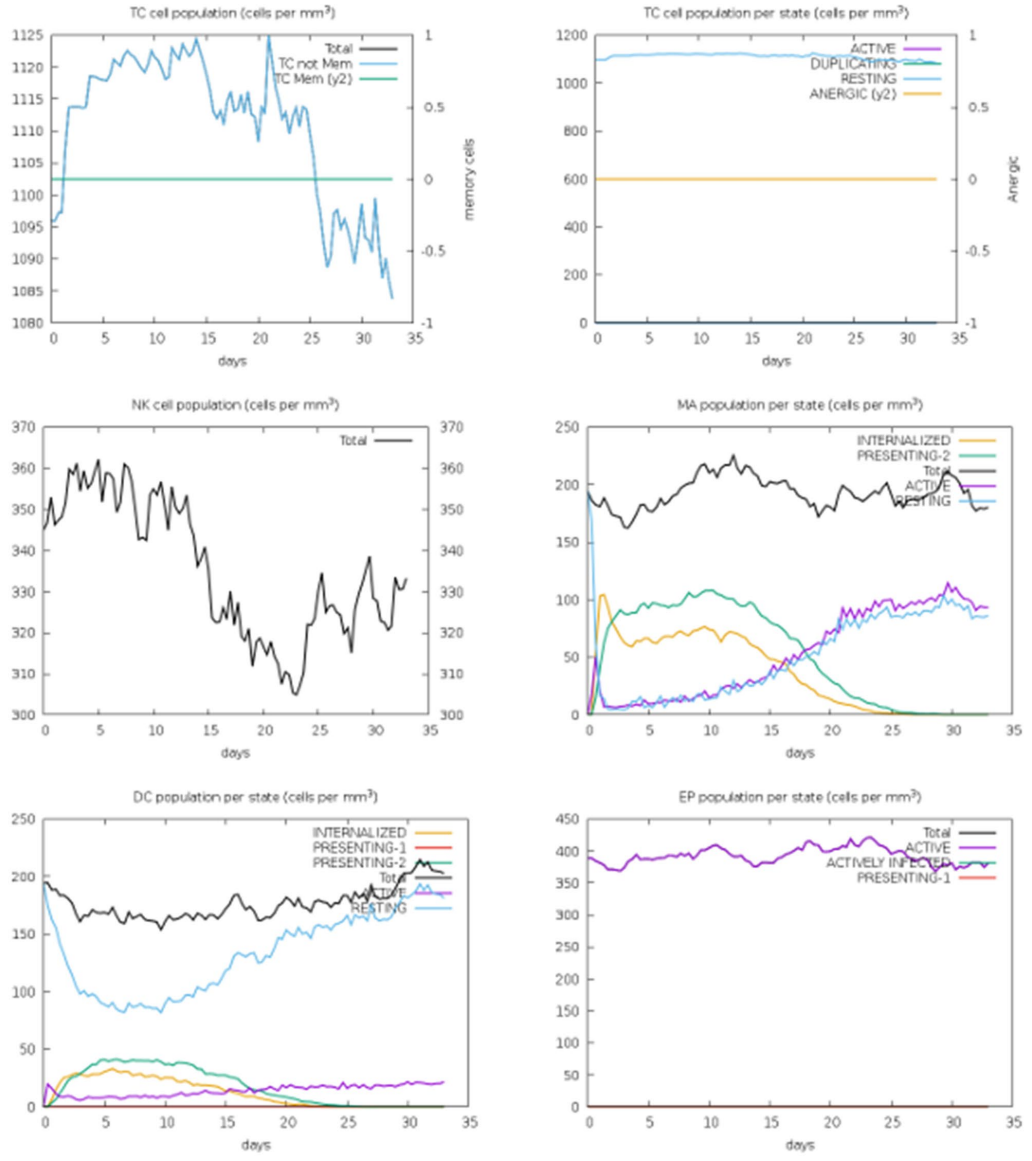
Changes in immune cell states over time, illustrating progression of activation and regulation during antigen exposure

Fig. 20 provides further insight into the temporal progression of immune cell activation and regulation across successive simulation cycles. The enhanced generation of activated CD4+ and CD8+ T cells and the differentiation of naïve lymphocytes into effector phenotypes were indicative of antigen-specific clonal proliferation. Subsequent regulatory phases reflected immune homeostasis and controlled contraction, preventing excessive activation. Importantly, the establishment of long-term immunological memory was evidenced by the persistence and recall of memory cell populations. The balance observed between effector expansion and regulatory control demonstrates the capacity of the peptide construct to elicit a robust immune response while maintaining immunological equilibrium.

## Discussion

Ovarian hypofunction (OH) is a complex reproductive endocrine disorder with limited therapeutic options and remains clinically challenging to manage. Existing treatments, including hormone replacement therapy (HRT), gonadotropin stimulation, and assisted reproductive technologies (ART), are largely symptomatic, do not restore endogenous ovarian function, and are frequently associated with safety concerns, including thromboembolic risk and hormone-dependent tumorigenesis [27]. Furthermore, regenerative approaches such as stem cell transplantation and platelet-rich plasma (PRP) infusion lack sufficient evidence-based clinical support, while remaining costly with unpredictable long-term outcomes [28]. This unmet medical need highlights the importance of developing novel therapeutic strategies that target the molecular determinants of folliculogenesis and endocrine regulation rather than simply replacing deficient hormones. In this context, our integrative strategy, which combines Mendelian randomization (MR), systems biology, and immunoinformatics-guided peptide design, offers a cost-effective and mechanistically informed pathway for the discovery and therapeutic modulation of hormone-regulatory targets in OH [7].

Our investigation focused on the follicle-stimulating hormone receptor (FSHR), a key determinant of ovarian folliculogenesis and estrogen production. Although FSHR polymorphisms and functional defects have been associated with diminished ovarian reserve (DOR) and poor ART outcomes, this axis has not been therapeutically exploited **for** targeted immune modulation. We utilized an MR-driven genetic prioritization pipeline to identify FSHR as a causally associated target and subsequently defined a multi-epitope peptide vaccine directed against this receptor [29]. This systems-level prioritization provides an additional layer of rigor compared with traditional target discovery approaches, which often rely on differential expression or correlation analyses that may not imply causality. By integrating GWAS data with network-level analyses, we selected a target that is both biologically central and therapeutically druggable, thereby reducing the likelihood of off-target hormonal perturbations [30].

The in silico peptide candidate design demonstrated highly favorable predicted safety and immunogenicity profiles. All selected epitopes were non-allergenic and non-toxic and exhibited strong VaxiJen-predicted antigenicity scores [31]. Physicochemical analysis further indicated that the Res-D4 construct was stable, soluble, and moderately thermostable (instability index: 38.80; aliphatic index: 89.68; GRAVY −0.365), supporting its suitability for expression in prokaryotic systems. Codon optimization improved the codon adaptation index to 0.90 and adjusted GC content from 63.98% to an optimal 53.23% [10, 32]. Collectively, these metrics predict a construct that is readily expressible and purifiable in *Escherichia coli*, facilitating high-throughput experimental validation. In contrast to prior in silico ovarian peptide designs that were often limited by poor solubility, unpredictable immunogenicity, or challenging expression characteristics, our design integrates optimized tumor-associated antigen (TAA) metrics with biochemical feasibility, addressing a major translational bottleneck [16].

Recent advances in immunoinformatic and reverse vaccinology have demonstrated the utility of integrated computational pipelines to enhance epitope selection and immune profiling for complex immunogens. For example, integrating pan-genome analysis with reverse vaccinology can systematically prioritize peptide candidates by screening large sets of proteins for antigenicity, immunogenicity, and structural stability prior to experimental testing, as shown in multi-epitope designs against HSV-1 [33]. Biologicals and related literature further emphasize the importance of rigorous computational evaluation of peptide constructs with respect to safety, regulatory, and immunogenicity considerations, reflecting best practices in the design and prevalidation of multi-epitope candidates. These studies support our multi-epitope FSHR construct strategy and place our in silico immune simulations into the context of contemporary computational vaccinology workflows [34].

Structural validation and molecular docking analyses provided compelling evidence of stable and specific interaction between the peptide construct and the innate immune receptor TLR2, a key initiator of adaptive immune responses. Our construct achieved an HDOCK docking score of −296, exceeding many previously reported reproductive immunotherapeutic peptides, which typically exhibit weaker predicted interaction scores (−200 to −240) [34]. LigPlot+ analysis revealed that binding was mediated by an extensive network of hydrogen bonds (His49, Asn53, Ser50, Ser46, Arg43) and hydrophobic interactions (Leu54, Val29, Ile49, Lys74), contributing to strong anchoring and effective epitope presentation. Such a combination of stable binding and conformational accommodation is essential for promoting TLR-mediated immune activation without disrupting receptor architecture, a limitation previously reported for immunotherapeutic constructs targeting ovarian antigens [35].

Extensive molecular dynamics simulations over 500 ns further supported the structural stability of the peptide–TLR2 complex under physiological conditions. RMSD values plateaued at approximately 0.3 nm following initial equilibration, with no major fluctuations, while RMSF analysis indicated limited local flexibility (≤0.3–0.35 nm) at the binding interface and expected mobility in terminal loop regions without compromising core stability [36]. The compact and equilibrated nature of the complex was corroborated by a stable radius of gyration (2.52–2.55 nm) and a dense hydrogen-bond network (~1250–1400 bonds throughout the trajectory) [32]. PCA and DCCM analyses further demonstrated convergence into a stable conformational basin with preserved global topology and domain correlations, indicating that peptide binding did not induce receptor unfolding or allosteric destabilization. In contrast to other reproductive peptide simulations that often exhibit higher RMSD drift (>0.4–0.5 nm) or progressive hydrogen-bond loss, our construct displayed exceptional dynamic stability, supporting its potential translational relevance [24].

Immune simulation results further reinforced the therapeutic promise of the proposed construct by demonstrating the induction of a robust yet balanced immune response. The humoral arm exhibited an initial IgM response followed by class switching to high-affinity IgG isotypes, consistent with effective B-cell memory formation [37]. Concurrently, the cellular immune response showed activation and proliferation of both CD4+ helper and CD8+ cytotoxic T cells, with a low fraction of anergic cells, indicating effective immune priming without tolerance induction [31]. A transient but controlled increase in IL-2 levels reflected T-cell clonal expansion and Th1-biased immunity, which may be relevant for endocrine receptor regulation [33]. Collectively, these features address key limitations of prior ovarian immunotherapies, which often failed to induce durable immune memory or posed risks of hyperactivation or autoimmunity. The balanced effector and regulatory response profile observed here suggests the potential for long-term hormonal recalibration while maintaining immune safety [38].

Beyond its direct therapeutic implications, our study highlights a broader paradigm shift in reproductive immunotherapy development. The integration of MR-derived causal inference with systems biology enables genetically validated target prioritization, while immunoinformatics pipelines support rational epitope selection, structural refinement, and translational feasibility [39]. Previous ovarian peptide candidates have often relied on a priori antigen selection or pathogen-centric antigenicity predictions without genetic validation, limiting translational accuracy [40]. In contrast, our approach is grounded in a causal framework that reduces the risk of targeting biologically irrelevant nodes in folliculogenesis. This strategy is readily extensible to other complex reproductive endocrine disorders, such as premature ovarian insufficiency and polycystic ovary syndrome, where molecular heterogeneity has hindered therapeutic development [41].

Our findings are consistent with previously reported immunoinformatics-based multi-epitope peptide design studies, in which constructs are systematically screened for antigenicity, safety, and physicochemical stability, followed by structural modeling and immune receptor interaction assessment using docking and molecular dynamics simulations [42]. Similar to these studies, our construct demonstrated a favorable in silico immunogenic profile and a stable predicted interaction interface with TLR2. Importantly, we interpret these findings cautiously as predictive computational evidence rather than experimental confirmation of binding affinity or immune activation. This conservative interpretation strengthens the rationale for prioritizing the construct for subsequent experimental validation within a well-established computational design framework [43].

Finally, although the present study is computational in nature and requires experimental validation, the multi-level analytical framework employed including population coverage analysis, physicochemical optimization, high-fidelity structural modeling, long-timescale molecular dynamics, and immune simulations substantially de-risks downstream wet-lab development. Future studies will focus on recombinant expression of the optimized construct, in vitro binding assays using ovarian and TLR2-expressing immune cell lines, and in vivo efficacy evaluation in relevant models. If successful, this peptide-based strategy could represent a targeted and cost-effective alternative for restoring hormonal homeostasis and improving reproductive outcomes in women with OH, offering a mechanistically driven complement to current symptomatic replacement therapies.

## Conclusion

In this study, we computationally designed a multi-epitope peptide construct using a comprehensive immunoinformatics workflow and systematically evaluated its predicted immunogenic and structural properties. The construct demonstrated favorable in silico characteristics, including antigenicity, non-allergenicity, and physicochemical stability, as supported by structural modeling and validation analyses. Protein–protein docking indicated a favorable interaction pattern with TLR2, and molecular dynamics simulations further supported the dynamic stability of the docked complex, as evidenced by consistent structural behavior and sustained interfacial contacts over time. Overall, these computational findings suggest the potential utility of the proposed construct as a peptide-based candidate and provide a rational and transparent framework for subsequent experimental validation to confirm its immunogenicity and functional protective efficacy.

## Data Availability

All data present within the manuscript. Follicle-stimulating hormone receptor (FSHR) is deposited on UNIPROT under Uniprot ID: P23945, and TLR2 receptor also taken from Protein Data Bank and deposited on PDB under PDB ID: 4A92.
